# Safety and Field Performance of a Monovalent Vaccine Against Variant *Moritella viscosa* in Atlantic Salmon Under Commercial Conditions

**DOI:** 10.3390/vaccines14050403

**Published:** 2026-04-30

**Authors:** Anette Furevik, Hege Jørstad Sekkenes, Bjørn Ellingsen, Ane Sandtrø, Svein Alexandersen, Øyvind Tønnessen, Binoy Rajan, Lars Gaute Jørgensen, Ingrid Margrethe Hagerup, Lisa Marie Leknes, Siv Haugen Tunheim, Ida Steine Oma, Christél Teie, Jostein Mulder Pettersen, Monica Gausdal-Tingbø

**Affiliations:** 1 PHARMAQ AS, Skøyen, P.O. Box 267, 0213 Oslo, Norway; 2 Salmalytics AS, Ullevålsveien 68, Bygning 23, 0454 Oslo, Norway

**Keywords:** *Moritella viscosa*, *Atlantic salmon*, fish vaccine, field trial, winter ulcer disease, aquaculture

## Abstract

**Background/Objectives:** This article reports results from a multisite field trial conducted as part of the regulatory evaluation of a novel monovalent vaccine for *Atlantic salmon* targeting an emerging variant of *Moritella viscosa*, with the objective of assessing safety and performance under commercial production conditions. **Methods:** The trial included approximately 8 million *Atlantic salmon* reared at seven freshwater and eleven seawater sites in Norway. At each site, fish in the test and control groups received identical vaccination regimens, with the test group additionally receiving the test vaccine. Fish were monitored from vaccination until harvest. Safety endpoints included post-vaccination mortality and local reactions; effectiveness endpoints included outbreak-related mortality and antibody responses. Harvest quality was evaluated as an exploratory endpoint. **Results:** Post-vaccination mortality within 21 days was low and comparable between groups. Local reaction scores were within acceptable ranges but slightly higher for the test group. Outbreaks of winter ulcer disease caused by variant *M. viscosa* occurred at three seawater sites, during which test groups were associated with substantially lower mortality compared with controls. At these sites, the proportion of downgraded fish at slaughter was consistently lower in test than control groups. Across sampling points, the test group showed higher antibody titers than the control group. **Conclusions:** Co-administration of the monovalent variant *M. viscosa* vaccine with commercial core vaccines was associated with reduced disease burden during outbreaks and a favourable safety profile under commercial farming conditions. These findings support its potential relevance in vaccination programmes for *Atlantic salmon* farming.

## 1. Introduction

Winter ulcer disease poses a persistent health and production challenge to *Atlantic salmon* (*Salmo salar*) aquaculture, particularly in Norway. The disease is characterised by superficial skin lesions that may progress to chronic ulcers and septicaemia, contributing to substantial animal welfare concerns and economic losses [1,2]. Outbreaks occur predominantly during the winter months, typically at seawater temperatures below 8 °C [3].

The causative agent, *Moritella viscosa*, was historically regarded as a relatively homogeneous bacterial species. However, over the past decade, investigations have identified emerging subpopulations with distinct genotypic and phenotypic traits [4]. One such group, referred to as variant *M. viscosa*, has been implicated in several outbreaks and displays limited antigenic similarity to the classic *M. viscosa* strain [5]. More recently, an additional group exhibiting altered colony morphology but retaining classical genetic features—termed classic non-viscous *M. viscosa*—was reported during outbreaks in the winters of 2021–2022. Antigenic analyses have shown that classic non-viscous isolates share key features with variant strains [6].

Farmed salmon in Norway are vaccinated intraperitoneally (IP) with multivalent core vaccines, which typically include a bacterin from classic *M. viscosa*. However, previous reports of winter ulcer outbreaks raised concerns about the level of protection provided by the vaccines available at the time [7]. Controlled laboratory challenge trials have shown that vaccines based on the classic *M. viscosa* strain provide poor protection against variant [7,8] and classic non-viscous *M. viscosa* isolates [6], confirming the need for antigenic matching between vaccine components and circulating field strains.

Experimental vaccines incorporating formalin-inactivated variant *M. viscosa* antigens have demonstrated efficacy in reducing ulcer development and mortality in controlled laboratory trials following challenge with variant [7,8] and classic non-viscous isolates [6]. However, these prototype vaccines have provided only suboptimal protection against classic *M. viscosa*, underscoring the strain-specific nature of vaccine-induced protection [7]. Together, these findings suggest that effective vaccination strategies should include both classic and variant *M. viscosa* antigens.

While pre-clinical laboratory trials provide essential data on immunogenicity and protection under controlled conditions, results from such studies may not fully reflect vaccine performance in commercial farming due to varying environmental and biological factors. Regulatory approval and industry adoption of novel vaccines therefore require validation under field-relevant production settings. Accordingly, this trial evaluated the safety and performance of a newly developed monovalent oil-adjuvanted vaccine targeting variant *M. viscosa* under commercial farming conditions in Norway, as part of the documentation required for regulatory approval.

## 2. Methods and Materials

### 2.1. Trial Design

This multisite field trial was designed to evaluate the safety and performance of a test vaccine in *Atlantic salmon* (*Salmo salar*) and was conducted in accordance with the principles of Good Clinical Practice (GCP) for veterinary field trials [9]. The trial population was followed from vaccination through to harvest.

The trial included seven freshwater vaccination sites and eleven seawater grow-out sites. The production structure followed a hierarchical relationship, whereby a single freshwater facility could supply fish from separate vaccination events to multiple seawater sites and cages. Each production unit was assigned a Unique Identity Code (UIC) consisting of three components: freshwater facility (FWx), vaccination cohort (Cy), and seawater site (SWz). Thus, a complete UIC (e.g., FW01–C1–SW01) identifies fish originating from freshwater site FW01, belonging to cohort C1, and subsequently transferred to seawater site SW01.

A cohort was defined as all fish vaccinated together in a single operation at the same freshwater site within the same time period using comparable vaccine batches. Multiple cohorts could be produced at each freshwater facility. Trial sites were geographically distributed along the Norwegian coastline, from Vestland county in the south to Finnmark county in the north, and all seawater sites had a history of winter ulcer outbreaks. One seawater site was excluded from the trial due to a severe parasitic infection resulting in high mortality unrelated to the trial vaccines.

Approximately 4.2 million fish received the test vaccine co-administered with commercially available core vaccines, while approximately 4.1 million fish received only the core vaccines and served as the control group. At each site, the control group received an identical vaccine regimen to the test group except for the omission of the test vaccine. In freshwater, test and control groups were held in separate tanks.

Following vaccination, fish were transferred by wellboat to their designated seawater sites according to commercial production plans. At each seawater site, test and control groups within cohorts were randomly allocated to separate cages of uniform size and design, each containing approximately equal numbers of fish. All seawater cages were standard circular sea cages used in commercial *Atlantic salmon* production. Each seawater site contained an equal number of test and control cages, except at two sites (SW07 and SW08), as described in Section 2.2.

Cohort FW07–C1–SW10 included six unique vaccination groups representing different combinations of core vaccines with or without the test vaccine. Fish were individually marked for identification, and the groups were distributed across six sea cages. Within each cage, two groups were co-stocked at an 80/20% ratio to ensure shared rearing conditions while maintaining clear group distinction.

### 2.2. Population

The study population consisted of commercially reared *Atlantic salmon* (*Salmo salar*) managed according to standard husbandry practices at the participating sites within the Norwegian regulatory framework.

Inclusion criteria: Eligible groups were required to be >25 g in weight, of uniform size, of the same population and genetic origin, and to have a documented health history supported by a veterinarian-issued health certificate. Fish had to be unvaccinated by injection (IP) prior to enrolment and clinically healthy at the time of vaccination. At the time of vaccination, at least 100 fish per group were weighed and 30 fish were examined for abdominal findings to document compliance with the minimum weight criterion and the absence of prior intraperitoneal vaccination.

Exclusion criteria: Fish displaying signs of injury, deformities, or sexual maturation were excluded at the time of vaccination. Fish that died due to technical procedures during or immediately after vaccination (e.g., anaesthesia or mechanical injury) were recorded but excluded from the mortality dataset. Mortality exceeding 0.5% within 21 days post-vaccination was predefined as an adverse event and triggered follow-up investigation.

Identification and traceability: Test and control groups were held in tanks and sea cages, each assigned a unique unit number. All movements of fish groups between units during production were documented to ensure full traceability, including transfers during vaccination, seawater transport, treatment procedures (e.g., delousing), and delivery to slaughter. In cohort FW07, individual fish were marked during vaccination under anaesthesia by adipose fin removal (AF) or by shortening of the left (LM) or right (RM) maxilla. These marking techniques enabled reliable group identification while minimising impact on fish welfare.

### 2.3. Trial Vaccines and Administration

#### 2.3.1. Vaccines

The test vaccine was a monovalent formulation containing formaldehyde-inactivated antigens of variant *M. viscosa*, designated Alpha Ject Moritella (abbreviated AJM). The inactivated antigen suspension was emulsified in mineral oil to produce a water-in-oil vaccine. The batch used in the trial was manufactured and released in accordance with Good Manufacturing Practice (GMP) standards.

The test vaccine was co-administered with a commercially available multivalent vaccine and, at some sites, with additional monovalent vaccines targeting common bacterial and viral pathogens in Norwegian aquaculture. The vaccines used in the trial are listed in Table 1. An overview of vaccine groups and cage allocations across cohorts is provided in Table 2, while the specific vaccination combinations used in cohort FW07-C1-SW10 are summarised in Table 3.

#### 2.3.2. Administration

Vaccination was performed according to standard commercial procedures at each freshwater site, primarily using an automated vaccination machine (Maskon, Stjørdal, Norway). At site FW02, calibrated pistol-grip syringes (Henke-Sass Wolf GmbH, Tuttlingen, Germany) were used instead. Within each cohort, treatment assignment during vaccination was randomised at the group level. Fish were fasted for at least 24 h prior to vaccination to minimise the risk of intragastric injection. Fish were collected randomly from the holding tank, anaesthetized, and vaccinated intraperitoneally, except for Clynav, which was administered intramuscularly in accordance with the product instructions. Vaccination quality, including injection site and vaccine deposition, was routinely assessed in both test and control groups.

Following vaccination, the accumulated degree days prior to seawater transfer varied across cohorts (range: 343–1650 degree days; Supplementary Table S1), which also summarises between-group differences in vaccination timing, fish weight at vaccination, and immunisation period prior to seawater transfer. While the immunisation period was not consistently above the recommended minimum for all vaccine combinations, groups with immunisation periods below the recommended level were matched between test and control, except for FW03-C1. In this cohort, due to practical limitations, test and control fish were immunised for 360 and 453 degree days, respectively.

### 2.4. Monitoring and Data Collection

#### 2.4.1. Sample-Based Data Collection

Sampling was planned at five nominal time points for each cohort: baseline (prior to vaccination), one week before seawater transfer, 5 and 10 months post-vaccination, and at a pre-harvest time point (within four weeks before harvest). In practice, some sampling time points deviated from the planned schedule due to constraints inherent to commercial production. For each cohort, one rearing unit (tank or cage) per treatment group (test and control) was sampled repeatedly across all time points to maintain consistency. Fish were randomly collected within each selected unit to obtain samples representative of the group, within the practical constraints of commercial production.

Abdominal local reactions: Baseline assessments of local reactions were performed prior to vaccination, with subsequent evaluations conducted at all planned sampling points. Adhesions were scored using the Speilberg scale (0–6), ranging from no adhesions (score 0) to extensive and severe adhesions (score 6), based on the highest regional score [10]. Melanisation of the viscera and abdominal wall was scored separately (0–3) according to the sponsor’s internal guidelines. All assessments were performed by personnel blinded to the treatment group.

Blood plasma and serology: Blood was collected from the same fish used for local reaction assessments. Samples were drawn into heparinised vacutainer tubes (Greiner Bio, Kremsmünster, Austria), centrifuged to separate plasma, and transferred to CryoTube™ containers (Thermo Fisher, Waltham, MA, USA) labelled with the sampling date and cage number. Plasma samples were shipped on ice or dry ice to the sponsor’s laboratory and stored at −20 °C until analysis. A targeted subset of four cohorts (FW03-C1-SW03, FW06-C1-SW09, FW06-C2-SW09, and FW07-C1-SW10) was selected for enzyme-linked immunosorbent assay (ELISA) analysis. Three cohorts were included because they experienced clinical winter ulcer outbreaks during the seawater phase, whereas FW07-C1-SW10 was included to assess antibody responses in the cohort with the most complex vaccine co-administration setting. ELISA was performed as described by [7], with full protocol details provided in Supplementary Method S1. Briefly, plates were coated with inactivated *M. viscosa* antigen, plasma samples were analysed in two-fold dilution series starting at 1:200, and bound antibodies were detected using monoclonal anti-salmonid IgM antibody 4C10 [11], followed by an HRP-conjugated secondary antibody. Optical density was read at 490 nm, and the positive sample dilution was defined as the dilution giving an OD value of 0.5 after blank subtraction and normalisation.

Morphometrics (weight and length): Body weight and fork length were recorded from the same fish used for the above sampling procedures, with additional fish measured where applicable.

#### 2.4.2. Routine Monitoring and Health Observations

Mortality and temperature: Water temperature and daily mortality were recorded according to standard routines at each site. Dead and moribund fish were routinely inspected by the investigator or designee, and the presence of skin ulcers was documented. During periods of elevated mortality or adverse weather, when individual counting was not feasible, mortality was estimated according to standard aquaculture practices, using bulk weight and subsampled mean weight of dead fish.

Diagnostics: All health issues, diagnoses, and both planned and unplanned treatments were recorded. Suspected or confirmed disease events triggered diagnostic sampling and follow-up measures planned jointly by the Investigator, Monitor, and Study Director. Diagnostic analyses were performed by accredited laboratories (PHARMAQ Analytiq, PatoGen AS, or the Norwegian Veterinary Institute), using routine diagnostic methods appropriate for the suspected condition. For ulcer events, diagnostic bacteriology included culture of ulcer swabs on blood agar supplemented with 2% NaCl under standard incubation conditions. Suspected *Moritella viscosa* growth was subcultured for purity and confirmed by real-time qPCR, with subsequent typing to distinguish classic, variant, and classic non-viscous *M. viscosa*. Winter ulcer outbreak periods were classified by the Investigator based on laboratory findings together with clinical signs of ulceration and patterns of increased mortality. All episodes of increased mortality, regardless of cause, were reported as adverse events (AEs).

Harvest data: Grading reports from slaughterhouses were collected from sites with confirmed winter ulcer outbreaks. Harvested fish were classified as Superior or Production quality in accordance with industry standards.

### 2.5. Evaluation of Safety and Effectiveness

The study included two safety endpoints (not hierarchically ranked) and three effectiveness endpoints (primary, secondary, and exploratory).

#### 2.5.1. Vaccine Safety Endpoints

Acute toxicity: Fish were observed daily for 21 days post-vaccination for abnormal behaviour, morphological changes, mortality, and other clinical signs. Mortality exceeding 0.5% within this period was predefined as an adverse event, in line with established vaccine safety criteria for *Atlantic salmon*. Fish that died due to anaesthesia or technical procedures during vaccination were recorded but excluded from the mortality analysis.

Local reactions: Abdominal adhesions and melanisation of the viscera and abdominal wall were scored at all post-vaccination sampling points to assess local tissue responses to vaccination.

#### 2.5.2. Vaccine Effectiveness Endpoints

Primary endpoint—mortality during disease outbreaks: Effectiveness was assessed by comparing cage-level mortality rates between test and control groups during confirmed outbreaks of winter ulcer disease caused by variant *M. viscosa*. Outbreak duration was defined by the site investigator based on clinical and diagnostic findings, and all diagnoses were verified by an accredited laboratory.

Secondary endpoint—antibody response: In a targeted subset of cohorts, antibody levels against variant *M. viscosa* were measured by ELISA to evaluate vaccine-induced immunogenicity and duration of response. For each group and sampling point, plasma samples from 30 fish were pooled (5 fish per pool; 6 pools per group) and analysed.

Exploratory endpoint—harvest quality data: Slaughter grading was used as an indirect indicator of performance and vaccine-associated effects at sites experiencing winter ulcer outbreaks caused by variant *M. viscosa*. Harvest data were summarised descriptively, as variation in slaughter timing—reflecting site-specific health status and operational harvesting constraints—and differing grading procedures between harvest facilities precluded formal statistical comparison.

### 2.6. Data Analyses

Statistical analyses of safety endpoints were performed to support interpretation of the field observations, although these analyses were not pre-specified in the study protocol. Estimated marginal means with 95% confidence intervals were obtained using the emmeans package (version 1.11.1). Standard model diagnostics indicated adequate model fit and stable parameter estimates. All statistical analyses were performed in R (https://www.R-project.org/) [12].

Acute toxicity: Mortality within 21 days post-vaccination was analysed using a negative binomial regression mixed model with a log link (glmmTMB package version 1.1.13). Treatment group was included as a fixed effect, the number of vaccinated fish as an offset, and cohort as a random intercept to account for variation between vaccination events. Estimated marginal means were obtained on the response scale.

Local reactions: Local reaction data from all cohorts except FW07-C1-SW10 were combined (*n* = 1199 test, *n* = 1190 control). In FW04-C1-SW04, one sampling (*n* = 30) was excluded because it was a late control-only sampling collected outside the planned sampling schedule, after slaughter had started, from residual size-sorted fish that was considered unrepresentative of the original group. Each ordinal endpoint (adhesions, visceral melanin, abdominal wall melanin) was analysed separately using cumulative link mixed models (ordinal package version 2023.12-4.1). Treatment group and sampling time point (pre-sea, 5 months post-vaccination (mpv), 10 mpv, pre-harvest) were included as categorical fixed effects. Cohort was modelled with a random intercept and a random slope for time to account for between-cohort variability in baseline levels and temporal trends, and study group nested within cohort was included as a random effect to account for non-independence of observations within the same group. A treatment-by-time interaction was evaluated using likelihood-ratio tests (LRT) and retained when *p* < 0.05. The proportional-odds assumption was assessed using cumulative link models without random effects; no violations were detected for the treatment effect. Apparent deviations for the time effect were attenuated after including random slopes, indicating that these reflected between-cohort heterogeneity rather than true non-proportionality. Model-based marginal category probabilities were used to construct stacked bar plots of predicted score distributions.

FW07-C1-SW10 was analysed separately to assess whether the addition of AERM to AJM increased tissue reaction severity. The cohort comprised four cages with two cohabitant groups per cage; analyses were restricted to cages 1 and 4, each containing two treatment groups (AJM vs. AJM + AERM; *n* = 90 per group). Because each cage used a different core vaccine, treatment and core vaccine were fully confounded, and each cage was analysed separately. The ordinal endpoints were analysed using cumulative link models (ordinal package) with treatment group and sampling time point as fixed effects. The treatment-by-time interaction was evaluated using LRT and retained when *p* < 0.05. For adhesions in cage 4, where only two score levels were observed, a bias-reduced logistic regression model was used (brglm2 package version 1.0.0). Potential deviations from the proportional-odds assumption and occasional convergence issues were examined using nominal tests and, where indicated, bias-reduced partial proportional-odds models using the same package. These sensitivity analyses yielded results consistent with the primary models.

Mortality during disease outbreaks: Clinical winter ulcer outbreaks occurred at three sea sites, each experiencing one outbreak in the first winter and one in the second winter of the seawater production phase. Sea-cage mortality during confirmed winter ulcer outbreaks was analysed using a negative binomial generalised linear mixed model with a log link (glmmTMB package). Delousing during the outbreak period occurred only at SW09, where all cages received one treatment during the second outbreak. Because delousing was completely confounded with site, it was not included in the mortality models. The number of dead fish per cage during each outbreak served as the response variable. An offset equal to the logarithm of fish-days (number of fish × outbreak duration) was used to adjust for differences in population size and time at risk. Partial harvesting at one site (SW03) required a time-weighted average stock count, whereas full harvesting of some cages at SW09 was accounted for directly through their shorter exposure period in the fish-days calculation. Treatment group was included as a fixed effect. Random intercepts were included for farm, with outbreak nested within farm (Farm/Outbreak). A treatment-by-outbreak interaction and a cage-level random intercept were evaluated but did not improve model fit and were not retained. A sensitivity analysis using fixed outbreak strata (Farm × Outbreak) yielded comparable treatment effect estimates.

Estimated marginal means and corresponding rate ratios with 95% confidence intervals were obtained from the primary model. Outbreak-specific estimates were derived from a model including the treatment-by-outbreak interaction for descriptive comparison between outbreak periods.

Antibody titers: Titer data were log_10_-transformed prior to analysis. For cohorts FW03-C1-SW03, FW06-C1-SW09, and FW06-C2-SW09, which experienced winter ulcer outbreaks, a linear mixed-effects model (nlme package version 3.1-164) was fitted with treatment group and sampling time point as categorical fixed effects and random intercepts for cohort and study group nested within cohort (*n* = 72 test; *n* = 72 control). A varIdent structure accounted for heterogeneous residual variances across time points. A treatment-by-time interaction did not improve model fit and was excluded. The final model was refitted using restricted maximum likelihood (REML). Estimated marginal means were back-transformed to yield geometric mean ratios (GMR; Test/Control) with 95% confidence intervals.

FW07-C1-SW10, which did not experience a winter ulcer outbreak, was analysed separately to assess whether the addition of AERM to AJM affected antibody titers. Generalised least-squares models (nlme package) were fitted to log_10_-transformed titers, with treatment group (Core + AJM and Core + AJM + AERM; *n* = 24 per group) and sampling time point (pre-sea, 5 mpv, 10 mpv, pre-harvest) as fixed effects. Interaction and heteroscedastic variance structures were evaluated but did not improve model fit. The final model included only main effects and was fitted using REML.

## 3. Results

### 3.1. Safety Endpoints

#### 3.1.1. Acute Toxicity

No abnormal behaviour or morphological changes were observed in any treatment group at any site during the 21-day acute toxicity period. Mortality remained below the predefined adverse-event threshold of 0.5% for all groups, except in one test group at FW01–C1, where a technical malfunction of the vaccination machine resulted in elevated mortality. This event was judged unrelated to the trial vaccines, and the cohort was therefore excluded from the statistical analyses.

Across the remaining cohorts, mortality was low and comparable between groups. The estimated mean mortality within 21 days post-vaccination was 0.156% (95% confidence interval (CI): 0.098–0.249) in the control group and 0.159% (95% CI: 0.100–0.252) in the test group. The test group showed a small, non-significant 2% increase in mortality risk relative to the control group (rate ratio = 1.02; *p* = 0.92), providing no evidence of treatment-related acute toxicity.

#### 3.1.2. Local Reactions

Local reaction data from all cohorts, excluding FW07-C1-SW10, were analysed jointly. The group × time interaction was not supported for any of the endpoints (adhesions: *p* = 0.10; visceral melanin: *p* = 0.70; abdominal wall melanin: *p* = 0.37). For adhesions, the overall odds ratio was 2.0 (95% CI 1.6–2.6, *p* < 0.001). For visceral melanin, the overall odds ratio was 1.5 (95% CI 1.2–1.9, *p* < 0.001). For abdominal wall melanin, the main group effect was not supported (OR = 1.1, 95% CI 0.8–1.5, *p* = 0.41). Overall, across all cohorts and endpoints, local reaction scores were slightly higher in test groups receiving AJM than in control groups.

**Figure 1:**
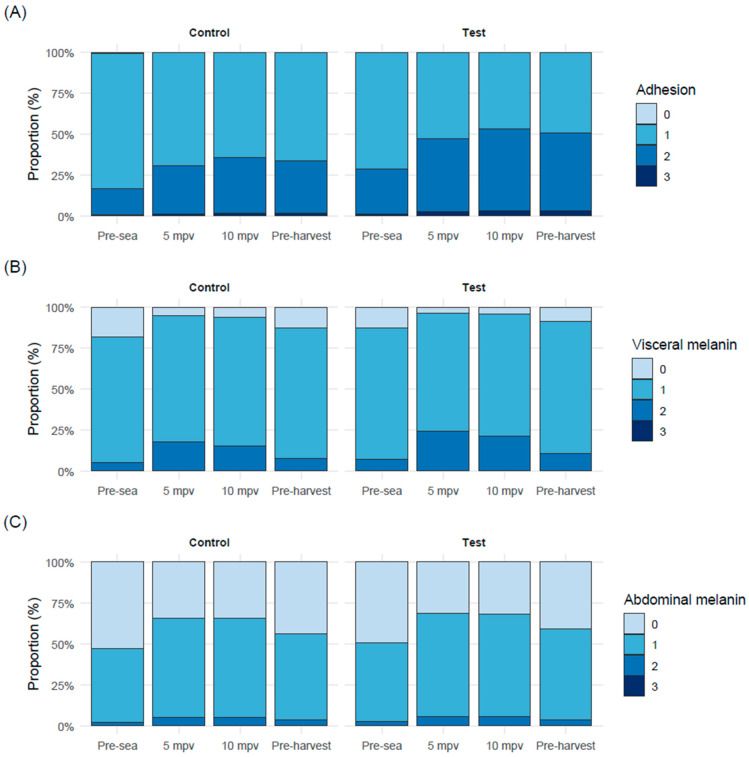
Stacked bar plots showing model-based predicted probabilities (from cumulative link mixed models) for each ordinal local reaction score across sampling time points. Panels (**A**–**C**) show Adhesions, Visceral melanin, and Abdominal wall melanin, respectively.

Model-based predicted score distributions are shown in Figure 1.

Analyses of local reaction scores in FW07-C1-SW10 found no evidence that the addition of AERM to AJM increased the severity of local tissue reactions. In cage 1 (AJm6), ordinal models indicated no differences between AJM + AERM and AJM for adhesions or visceral melanin, while abdominal wall melanin scores were marginally higher for AJM + AERM (OR = 2.2, 95% CI 1.0–4.6; *p* ≈ 0.05). In cage 4 (AJm7), logistic and ordinal models revealed no consistent group differences. Overall, these analyses indicate that the addition of AERM did not produce consistent or biologically meaningful differences in local reaction scores in this cohort.

No exceptionally high or atypical local reactions were observed, and adhesion scores did not exceed 3 on the Speilberg scale (0–6) in any cohort.

### 3.2. Effectiveness Endpoints

#### 3.2.1. Mortality

Clinical outbreaks of winter ulcer disease caused by variant *M. viscosa* occurred at three sea sites (SW03, SW07 and SW09). All three sites experienced an outbreak during both the first and second winter of the seawater production phase. Specific sampling was conducted following observations of increased mortality and/or ulceration at each site, and diagnoses were verified by an accredited laboratory. Outbreak periods were defined by the site investigators.

**Figure 2:**
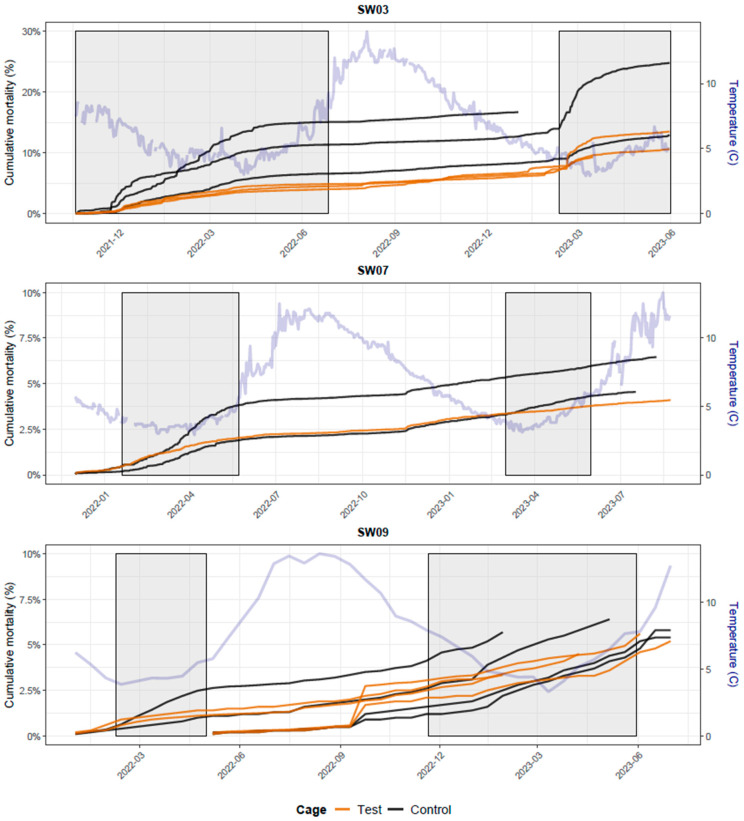
Cumulative mortality (%) over the full production period in individual sea cages at the three seawater sites experiencing winter ulcer outbreaks. The *x*-axis shows calendar time (year–month), the left *y*-axis shows cumulative mortality as a percentage of stocked fish, and the right *y*-axis shows seawater temperature (°C). Each orange line represents one test cage and each black line one control cage. The violet line shows seawater temperature over time. Shaded grey areas denote the first and second outbreak periods, defined based on increases in mortality associated with confirmed winter ulcer diagnoses.

Cumulative mortality during the outbreak periods differed among sites and between the first and second outbreaks (Figure 2). SW03 showed the highest cumulative mortality in both outbreak periods, whereas mortality remained lower at SW07 and SW09. Differences in time at risk during the outbreak periods reflect both variation in outbreak duration and shorter follow-up where cages were harvested during the outbreak period.

At SW03, mean cumulative mortality across cages was 4.5% in the test group and 11.0% in the control group during the first outbreak period, and 4.2% and 9.3%, respectively, during the second. Time at risk per cage was 251 days in the first outbreak period and ranged from 34 to 110 days in the second. At SW07, the corresponding mortality values were 1.6% and 2.6% in the first outbreak period, and 0.5% and 0.9% in the second, with time at risk of 125 and 92 days, respectively. At SW09, mean cumulative mortality was 0.8% and 1.4% in the first outbreak period and 2.1% and 3.5% in the second, with corresponding time at risk of 82 days in the first outbreak period and 66–221 days in the second.

Across both outbreaks, cages receiving the test vaccine exhibited significantly lower mortality compared with control cages (rate ratio = 0.57, 95% CI 0.45–0.74; *p* < 0.001), corresponding to an estimated 43% reduction in mortality.

There was no evidence of heterogeneity in treatment effect between outbreak periods (LRT *p* = 0.79). Outbreak-specific estimates derived from the interaction model showed a 46% reduction during the first outbreak (rate ratio = 0.54, 95% CI 0.37–0.78; *p* = 0.001) and a 40% reduction during the second outbreak (rate ratio = 0.60, 95% CI 0.43–0.85; *p* = 0.003).

#### 3.2.2. Antibody Titers

In the combined analysis of cohorts that experienced winter ulcer outbreaks (FW03-C1-SW03, FW06-C1-SW09, and FW06-C2-SW09), no significant interaction between treatment group and sampling time was detected (*p* = 0.18). Across sampling points, the Test group showed consistently higher antibody titers than the Control group (*p* = 0.01), corresponding to a geometric mean ratio (Test/Control) of 9.9 (95% CI: 3.4–28.8). The treatment effect remained stable in leave-one-cohort-out analyses.

Cohort FW07-C1-SW10, which did not experience a winter ulcer outbreak, was evaluated separately. Antibody titers increased after sea transfer in both vaccinated groups and remained elevated through harvest, but no overall difference was detected between the Core + AJM and Core + AJM + AERM groups (GMR = 0.95, 95% CI: 0.65–1.40; *p* = 0.79). Titers in both vaccinated groups were descriptively higher than in the core-only reference group, which was held in a different sea cage and was therefore not included in the formal statistical comparison.

**Figure 3:**
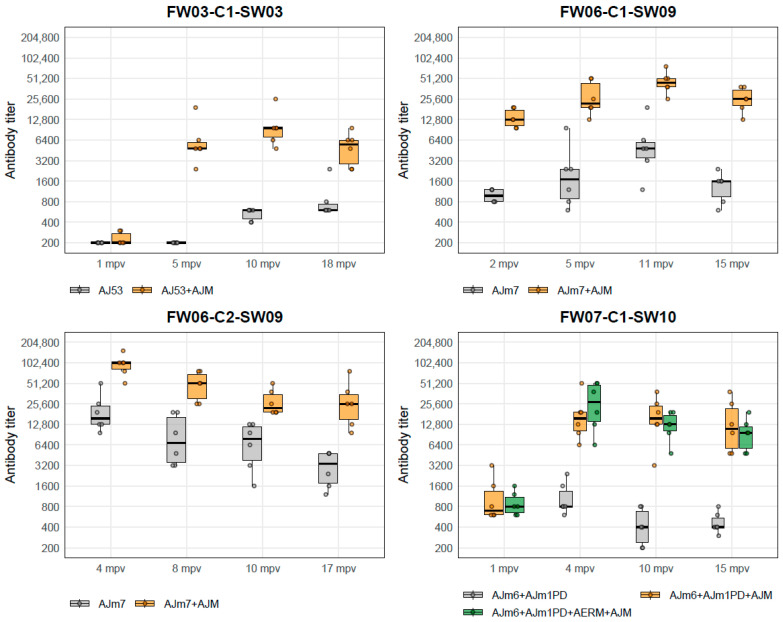
Distribution of antibody titers (reciprocal dilution) shown as boxplots with overlaid individual data points (dots) across the four sampling time points in each cohort. Orange denotes groups vaccinated with core vaccines supplemented with the test vaccine AJM, grey denotes core-only control groups, and green (FW07-C1-SW10 only) denotes the group receiving the core vaccine supplemented with both AJM and AERM. In FW07-C1-SW10, the grey control group was held in a different sea cage and is therefore shown for descriptive reference only.

The distribution of raw antibody titers for each cohort and sampling time point is shown in Figure 3.

#### 3.2.3. Harvest Quality

**Figure 4:**
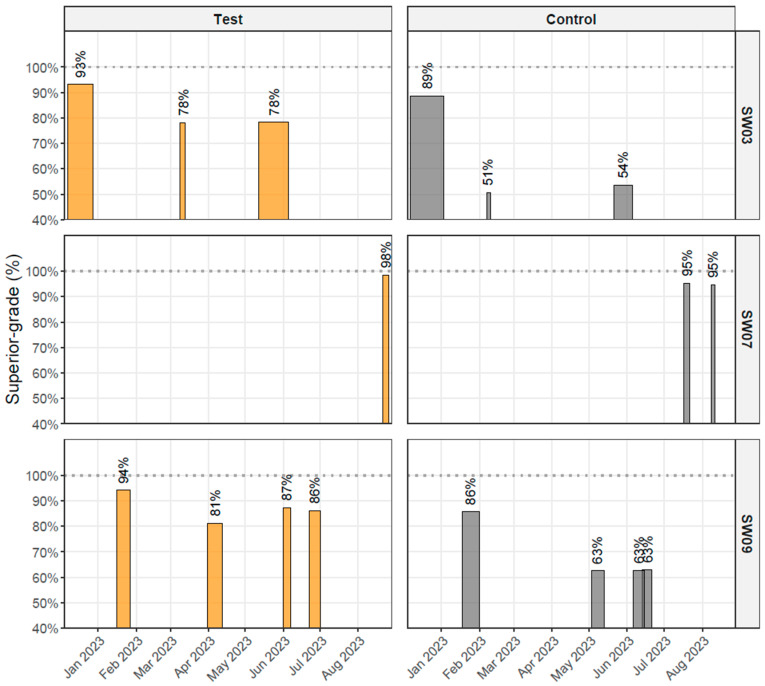
Harvest timelines and Superior-grade proportion by group across harvest events at sites SW03, SW07 and SW09. Bar width indicates the harvest period duration, and bar height the percentage of fish classified as Superior at harvest on a 0–100% scale (test: orange; control: grey).

Harvest grading results for treatment groups at sites experiencing winter ulcer outbreaks are summarised in Figure 4 and Supplementary Table S2. Across all outbreak sites (SW03, SW07 and SW09), the proportion of fish classified as Superior was consistently higher in the test groups than in the corresponding control groups. At the two sites where ulcer-related downgrading was recorded (SW07 and SW09), the proportion of fish downgraded due to ulcers was likewise lower in the test groups (Supplementary Table S2). The magnitude of these differences varied between cages and slaughter periods, reflecting biological variation (including differences in winter ulcer severity and other causes of downgrading), as well as differences in harvest timing within and between cages and in grading procedures across harvest facilities. Because such operational variation precluded meaningful statistical comparison, the results are presented descriptively. Overall, the observed trend of a higher proportion of Superior-grade fish in the test groups aligns with the lower outbreak-associated mortality observed in these cohorts.

## 4. Discussion

This field trial evaluated the safety and field performance of a monovalent variant *M. viscosa* vaccine administered in combination with commercial core vaccines under standard farming conditions. Across nine cohorts, the vaccine demonstrated a favourable safety profile and was associated with reduced disease burden during winter ulcer outbreaks, while serological analyses indicated a sustained antibody response in the evaluated cohorts.

During the 21-day toxicity period, mortality, abnormal behaviour, and morphological changes were closely monitored. No signs of vaccine-induced toxicity were observed, and post-vaccination mortality remained within the expected range for routine intraperitoneal vaccination of *Atlantic salmon*.

Local reactions were generally mild-to-moderate across all cohorts and remained within ranges considered acceptable in commercial salmon farming. A slightly higher prevalence of adhesions and melanisation was observed in the test groups, which is plausible given the greater total volume of oil-adjuvanted vaccine administered. The temporal pattern of reactions aligned with published descriptions, typically peaking around 6 months post-vaccination [13]. All reactions remained within the mild-to-moderate range, and no severe cases were recorded. This is relevant, as adverse effects such as growth impairment have been associated with high-severity local reactions [14,15]. In contrast, within the mild-to-moderate range, local reaction scores have generally not been shown to explain additional variation in growth beyond the general effect of vaccination [16,17].

Serological analyses indicated a clear and sustained specific antibody response relative to controls, with titers increasing after vaccination and remaining elevated throughout the seawater phase. A similar long-lasting antibody response has been reported in *Atlantic salmon* vaccinated with a multivalent vaccine co-injected with a monovalent vaccine, with elevated titers persisting throughout the 20-month production cycle [18]. The temporal pattern of responses was broadly consistent across cohorts, although pre-sea variation likely reflected differences in the interval between vaccination and sampling. The three cohorts included in the joint analysis experienced winter ulcer outbreaks, and natural exposure may have enhanced antibody levels in both groups, with unvaccinated fish expected to show larger proportional increases following infection [19]. In the cohort that did not experience an outbreak, the AJM-containing groups also showed high antibody titers, whereas the core-only reference group was descriptively lower. Although this comparison was descriptive only, the apparent separation between vaccinated and reference fish was greater than the Test/Control contrast estimated in the outbreak-affected cohorts. This observation is consistent with vaccinated fish maintaining high titers across cohorts, while natural exposure in outbreak-affected cohorts may have boosted titers in control groups, thereby reducing the relative difference between groups.

Across the winter ulcer outbreaks, the test groups consistently showed lower mortality than the control groups. Although the magnitude of the differences varied among sites, the direction of the effect was uniform across both outbreaks and is consistent with the persistent AJM-specific antibody titers observed in vaccinated fish. Slaughter data showed a similar pattern: at affected sites, the proportion of Superior-grade fish was higher in the test groups, whereas ulcer-related downgrading was lower when such data were available. Although the harvest results were descriptive due to variability in grading procedures, partial harvests, and differing harvest timings, the consistency of these findings is compatible with a lower overall disease burden in the test groups.

Several methodological aspects specific to the mortality endpoint should be considered. The analyses relied on total mortality rather than cause-specific mortality, as the latter was not recorded consistently and cause attribution under commercial field conditions can be imprecise [20,21]. Consequently, deaths unrelated to winter ulcer may have diluted group differences and biassed effect estimates towards the null. The onset and duration of outbreaks were defined at the site level according to protocol criteria, but cage-level disease dynamics can differ, and some cages may therefore have been misclassified with respect to the outbreak window, potentially biassing estimates in either direction. Moreover, changes in production plans resulted in an imbalance in the number of test and control cages at some sites. Such imbalance reduces the representativeness of the smaller group and makes its mortality estimates more sensitive to cage-specific conditions. In cohort FW03-C1, the immunisation period prior to seawater transfer was below the recommended level and shorter in the test group. This may have biassed the estimated group difference in a conservative direction, particularly given that an outbreak occurred shortly after transfer.

### Methodological Considerations

Several methodological aspects should be considered when interpreting the findings of this field trial. As is typical for large-scale studies conducted under commercial production conditions, the design was constrained by operational logistics. Although treatment assignment and allocation to production units were randomised, the study did not have the degree of balance and control achievable in a tightly controlled experimental design.

Because endpoints were mostly evaluated at the cage level, comparisons between test and control groups may reflect not only vaccine effects but also cage-specific factors such as variation in outbreak timing and intensity, hydrodynamic conditions, biomass density, fish weight, or concurrent health issues, potentially biassing estimates in either direction. Available health reports were reviewed descriptively, but they provided limited and non-standardised information on concurrent disease events and other possible contributors to mortality. Within these limitations, the available information did not indicate any clear alternative explanation for the observed mortality patterns, although non-detection may reflect differences in monitoring and diagnostic intensity rather than true absence. By contrast, delousing treatments were recorded at the cage level and by date. No delousing treatments were conducted during the outbreak periods except at SW09, where all cages were treated during the same time period. This is relevant because delousing procedures are considered a major source of mortality in commercial *Atlantic salmon* farming in Norway [22,23], reducing the likelihood that delousing acted as a differential confounder within outbreak periods. Although random error can be mitigated by a high number of observations, cage-level designs remain susceptible to systematic error and unmeasured confounding [24,25], introducing uncertainty into causal inference. Between-group differences in the timing of vaccination relative to the production cycle, including vaccination period, fish weight at vaccination, and immunisation time prior to seawater transfer, represent another potential source of confounding. However, such differences were not directionally consistent, making them unlikely to explain the direction of associations observed across endpoints (Supplementary Table S1). Importantly, the protective efficacy of the test vaccines against variant *M. viscosa* has been demonstrated in controlled challenge studies [7,8], supporting the biological plausibility of the field observations.

The sampling design also introduced certain limitations. For local reactions and antibody titers, only one test and one control cage were available per cohort, and scoring of adhesions and melanisation involved a degree of subjectivity, which may introduce measurement variability. Differences in sampling intervals, particularly at the pre-sea stage, may have contributed to early variation driven by time since vaccination rather than true biological differences. Importantly, test and control groups within each cohort were sampled concurrently, which reduces temporal confounding and strengthens the internal validity of within-cohort comparisons. On two occasions, sampling was conducted in a different fish group than in adjacent samplings; this was considered to have minimal impact because fish groups within a cohort are expected to follow similar trajectories. One cohort also deviated slightly from the main grouping structure because it was split across two sea sites after freshwater sampling. As this affected only a small subset of the data, site was not included as a separate random effect in the main local reaction analyses, and sensitivity analyses excluding this cohort did not materially alter the results. Obtaining fully representative samples from tanks and, in particular, from large sea cages is inherently challenging, and partial representativity may introduce selection bias [26]. Nevertheless, these datasets represent extensive collections of local reaction and antibody measurements under commercial conditions, and the statistical models accounted for between-cohort variation, although they cannot correct for systematic sampling differences.

The study was not designed to evaluate growth performance, and reliable inference on weight gain would have required large sample sizes, cohabiting groups, strict block randomisation with documented equal start weights, or Passive Integrated Transponder (PIT)-tagged designs that allow individual adjustment for baseline weight, as well as representative weight sampling across cages or slaughter periods [16,17]. These requirements are difficult to meet under large-scale commercial conditions, and formal growth analyses were therefore not undertaken. Routine production monitoring did not indicate abnormalities in growth, although precise evaluation of growth effects would require dedicated experimental designs.

Advances in camera-based monitoring may help address some of the practical constraints highlighted above. Such systems, capable of distinguishing marked fish and continuously quantifying lesions, growth and other parameters, offer the potential for high-resolution data that complement traditional sampling-based approaches and may strengthen future evaluations of vaccine performance under commercial farming conditions [27,28].

While the trial cannot, by design, match the internal validity of controlled experimental studies, it offers valuable external validity and complements challenge experiments in which protective efficacy against *M. viscosa* has been demonstrated. The present results should therefore be viewed as reflecting vaccine performance under real-world commercial conditions, with the above methodological considerations in mind.

## 5. Conclusions

This large-scale field trial found that a monovalent variant *Moritella viscosa* vaccine co-administered with commercial core vaccines was associated with reduced disease burden during winter ulcer outbreaks, while maintaining an acceptable safety profile under commercial farming conditions. These findings support further use and evaluation of the vaccine under commercial *Atlantic salmon* production conditions.

## Figures and Tables

**Table 1:** List of vaccines administered across the trial cohorts, including the test vaccine and the core vaccines used in each cohort.

Vaccine Type	Vaccine	Marketing Authorisation Holder	Abbreviation	Antigens	Injection Volume (mL)	Recommended Immunisation Period (Degree Days; SPC)
Test vaccine	Alpha Ject Moritella	PHARMAQ part of Zoetis	AJM	Variant *M. viscosa*	0.025	534
Multivalent vaccines	Alpha Ject micro 7 ILA		AJm7	*Aeromonas salmonicida, Vibrio anguillarum* O1/O2α, *Vibrio salmonicida*, classic *Moritella viscosa*, Infectious pancreatic necrosis virus (IPNV), Infectious salmon anaemia virus (ISAV)	0.05	520
	Alpha Ject 5-3		AJ53	*Aeromonas salmonicida*, *Vibrio anguillarum* O1/O2α, *Vibrio salmonicida*, classic *Moritella viscosa*.	0.1	520
	Alpha Ject micro 6		AJm6	*Aeromonas salmonicida*, *Vibrio anguillarum* O1/O2α, *Vibrio salmonicida*, classic *Moritella viscosa* and Infectious pancreatic necrosis virus (IPNV)	0.05	520
Monovalent vaccines	Alpha ERM Salar		AERM	*Yersinia ruckeri*	0.025	520
	Alpha Ject micro 1 PD		AJm1PD	Salmonid alphavirus (SAV3)	0.05	516
	Clynav	MSD Animal Health	Clynav	Salmonid alphavirus (DNA plasmid)	0.05	399

**Table 2:** Overview of trial sites and cohorts, including vaccine groups, number of sea cages per group, and the number of fish stocked at seawater transfer.

Unique Identity Code (UIC)			Group	Vaccine Combinations	No. of Cages	Total Number of Fish per Cage at Seawater Transfer
Freshwater Site	Cohort	Seawater Site (Production Area ^1^)				
FW01	C1	SW01 (12)	Test	AJm7 + AERM + AJM	1	112,218
			Control	AJm7 + AERM	1	149,853
FW02	C1	SW02 (3)	Test	AJ53 + Clynav + AJM	1	176,433
			Control	AJ53 + Clynav	1	177,212
	C2		Test	AJ53 + Clynav + AJM	1	199,709
			Control	AJ53 + Clynav	1	151,070
FW03	C1	SW03 (11)	Test	AJ53 + AJM	3	199,753
						199,847
						199,765
			Control	AJ53	3	199,308
						199,527
						199,376
FW04	C1	SW04 (12)	Test	AJm6 + AJM	4	110,126
						107,829
						109,275
						108,093
			Control	AJm6	4	107,737
						107,438
						106,413
						108,667
FW04	C2	SW05 (12)	Test	AJm6 + AJM	3	199,571
						195,634
						195,423
			Control	AJm6	3	195,710
						197,584
						198,217
FW04	C3	SW06 (12)	Test	AJm6 + AJM	2	184,843
						199,191
			Control	AJm6	2	191,213
						197,239
FW05	C1	SW07 (10)	Test	AJm7 + AERM + AJM	1	199,994
			Control	AJm7 + AERM	2	199,981
						199,982
		SW08 (10)	Test	AJm7 + AERM + AJM	3	130,829
						130,245
						131,324
			Control	AJm7 + AERM	1	183,113
FW06	C1	SW09 (8)	Test	AJm7 + AERM + AJM	2	152,200
						152,200
			Control	AJm7 + AERM	2	151,900
						152,500
	C2		Test	AJm7 + AERM + AJM	2	150,420
						149,380
			Control	AJm7 + AERM	2	149,930
						150,270

^1^ Numbers in parentheses indicate the Norwegian production area for the respective seawater site.

**Table 3:** Overview of vaccination combinations and cage design for cohort FW07-C1-SW10 (production area 6). Cages 1 and 4 were used to evaluate the add-on effect of AERM to the AJM-supplemented core vaccines, while cages 2, 3, 5, and 6 were used to assess the add-on effect of AJM to their respective core vaccines.

Cage No.	Vaccine Combinations	Marking *	Total Number of Fish per Cage at Seawater Transfer
1	AJm6 + AJm1PD + AJM	None	24,118
	AJm6 + AJm1PD + AJM + AERM	LM	6120
2	AJm6 + AJm1PD	AF	6412
	AJm6 + AJm1PD + AJM	None	23,796
3	AJm6 + AJm1PD	AF	24,065
	AJm6 + AJm1PD + AJM	None	6377
4	AJm7 + AJm1PD + AJM + AERM	None	23,919
	AJm7 + AJm1PD + AJM	RM	5990
5	AJm7 + AJm1PD	None	6151
	AJm7 + AJm1PD + AJM	RM	24,759
6	AJm7 + AJm1PD	None	24,442
	AJm7 + AJm1PD + AJM	RM	6043

* AF = adipose fin; LM = left maxilla; RM = right maxilla.

## Data Availability

The data that support the findings of this study are available upon reasonable request.

## Supplementary Materials

The following supporting information can be downloaded at: https://www.mdpi.com/article/10.3390/vaccines14050403/s1, Supplementary materials include additional tables supporting the study design (Supplementary Table S1) and harvest outcomes (Supplementary Table S2), as well as the ELISA protocol (Supplementary Method S1).
