# Supplementary material for: Safety and Field Performance of a Monovalent Vaccine Against Variant Moritella viscosa in Atlantic Salmon Under Commercial Conditions
Source: Vaccines (Basel). 2026 Apr 30;14(5):403. doi: 10.3390/vaccines14050403 (PMC13211632; doi:10.3390/vaccines14050403)
Supplement: Supplementary file 1 [file vaccines-14-00403-s001.zip › vaccines-4223606-supplementary.pdf]

Supplementary Table S1. Vaccination timing, fish weight at vaccination, and immunization period prior to seawater transfer by cohort and group.

			Date and number at vaccination		Weight at vaccination			Sea transfer		
**FW site**	**Cohort**	**Group**	**Period of vaccination**	**No. of vaccinated fish**	**Count (n)**	**Average**	**95% CI**	**SW site**	**Weeks post vaccination**	**Immunization period (degree days)**
FW01	1	Test	04.08-09.08.2021	454 705	100	48.3	46.4-50.3	SW01	14-15	>800
		Control	10.08-15.08.2021	477 326	200	42.3	41.2-43.4		11	>800
FW02	1	Test	29.06-01.07.2021	177 215	100	64.9	63.1-66.7	SW02	6	≥549
		Control	25.06-29.06.2021	178 930	100	64.5	62.9-66.1		6-7	≥561
	2	Test	13.09-15.09.2021	200 149	100	73.4	71.6-75.2		3	≥343
		Control	11.08-30.08.2021	151 388	100	99.6	96.6-102.6		2-5	≥356
FW03	1	Test	30.08-01.09.2021	600 279	100	65.3	62.8-67.8	SW03	6-7	≥360
		Control	23.08-26.08.2021	601 074	300	58.5	57.1-59.9		7-8	≥453
FW04	1	Test	25.10-27.10.2021	435 825	200	48.3	47.2-49.4	SW04	5	≥502
		Control	27.10-29.10.2021	432 383	200	45.9	44.8-47.0		5	≥514
FW04	2	Test	14.02-17.02.2022	592 104	100	47.4	46.0-48.8	SW05	10	≥727
		Control	21.02-09.03.2022	592 992	100	65.6	64.3-66.8		7-9	≥518
FW04	3	Test	14.02-17.02.2022	384 702	100	61.4	60.3-62.5	SW06	10	≥711
		Control	21.02-09.03.2022	388 901	100	42.6	41.4-43.9		7-9	≥606
FW05	1	Test	05.10-07.10.2021	601 211	100	57.7	55.6-59.8	SW07	8	≥612
								SW08	28-30	≥1650
		Control	05.10-07.10.2021	600 975	100	57.0	54.9-59.9	SW07	8	≥608
								SW08	28-30	≥1650
FW06	1	Test	11.10-12.10.2021	305 772	100	58.8	57.3-60.0	SW09	9	≥780
		Control	09.10-10.10.2021	306 137	100	59.7	57.9-61.5		9	≥1301
	2	Test	05.01-06.01.2022	300 596	100	85.5	84.0-87.0		17	≥780
		Control	04.01-06.01.2022	301 335	100	66.2	64.0-68.4		17	≥1301
FW07	1	AJm6+AJm1PD	21.06-24.06.2021	30 667	400	47.4	46.8-48.1	SW10	6	≥550
		AJm7+AJm1PD	21.06-24.06.2021	30 736					6	≥550
		AJm6+AJm1PD+AJM	21.06-24.06.2021	54 460	200	46.3	45.4-47.3		6	≥550
		AJm7+AJm1PD+AJM	21.06-24.06.2021	36 975					6	≥550
		AJm6+AJm1PD+AERM+AJM	21.06-24.06.2021	6 140					6	≥550
		AJm7+AJm1PD+ AERM+AJM	21.06-24.06.2021	24 035					6	≥550

Supplementary Table S2. Harvest grading results from sites experiencing winter ulcer outbreaks. Tables (A–C) correspond to sites SW03, SW07, and SW09, respectively

.

(A) Seawater site SW03 (UIC: FW03-C1-SW03)

	Test			Control		
	**Cage 1+5**	**Cage 7+5**	**Cage 1+11**	**Cage 3+7**	**Cage 3**	**Cage 3+9**
Slaughter period	07.12.22-27.12.22	09.03.23-12.03.23	12.05.23-04.06.23	07.12.22-02.01.23	07.02.23	21.05.23-04.06.23
No. of fish	92 715	214 756	218 126	176 188	71 218	199 566
Superior (%)	93.2	78.2	78.3	88.6	50.7	53.5
Production (%)	6.8	21.8	21.7	11.4	49.3	46.5
Downgrading due to ulcers	Not registered					

(B) Seawater site SW07 (UIC: FW05-C1-SW07)

	Test	Control	
	**Cage 11**	**Cage 10**	**Cage 12**
Slaughter period	21.08.23-25.08.23	08.08.23-10.08.23	17.07.23-21.07.23
No. of fish	151 366	148 634	156 587
Superior (%)	98.5	94.6	95.3
Production (%)	1.5	5.4	4.7
Downgrading due to ulcers	1.5	5.4	4.7

(C) Seawater site SW09 (UIC: FW06-C1-SW09 and FW06-C2 SW09)

	Test				Control			
	**C1**		**C2**		**C1**		**C2**	
	**Cage 1**	**Cage 4**	**Cage 9**	**Cage 10**	**Cage 2**	**Cage 3**	**Cage 7**	**Cage 8**
Slaughter period	16.01.23-26.01.23	31.03.23-11.04.23	22.06.23-30.06.23	01.06.23-06.06.23	18.01.23-31.01.23	03.05.23-12.05.23	13.06.23-20.06.23	06.06.23-14.06.23
No. of fish	141 327	146 531	140 333	142 729	147 310	139 035	141 059	138 833
Superior (%)	94.1	81.2	86.2	87.3	85.7	62.6	63.0	62.8
Production (%)	5.9	18.8	13.8	12.7	14.3	37.4	37.0	37.2
Downgrading due to ulcers	2.4	14.9	7.1	8.8	10.0	30.0	30.0	31.6

Supplementary Method S1

The ELISA procedure used in this study followed the method described by Furevik et al. (2023), with the full protocol summarized below.

ELISA was performed by coating poly-L-lysine (PLL)-precoated Nunc MaxiSorp (Thermo Fisher) 96-well plates with inactivated *M. viscosa* bacterial suspension diluted in 2% NaCl to a theoretical OD600 of 0.5, calculated from OD at harvest. Free binding sites were blocked with 5% skimmed milk in PBS containing 0.05% Tween 20 for 2 h at room temperature. A two-fold dilution series of each plasma sample, starting at 1:200, was added to the plate wells (100 uL per well). Two standard positive plasma sample pools from Alpha Ject Moritella-vaccinated salmon were included as positive controls to verify assay validity and as normalization controls for inter-assay adjustment. PBS was added as a blank sample. Plasma from mock (PBS)-vaccinated fish was included as a negative control. The negative control and normalization control were added at fixed dilution, whereas the positive control was treated similarly to the samples. Plasma samples and controls were incubated overnight at 2-8 °C.

A monoclonal antibody, 4C10 (Thuvander et al., 1990), diluted 1:3500 in PBS containing 1% dry skimmed milk and 0.05% Tween 20, was used for primary detection of circulating IgM. A horseradish peroxidase (HRP)-conjugated rabbit anti-mouse Ig secondary antibody (Thermo Fisher) was then added, followed by SigmaFast OPD substrate (Sigma) according to the manufacturer's recommendation. The OPD reaction was developed for 20 min in the dark at room temperature and stopped by addition of 1 M H2SO4. Optical density was read at 490 nm using a BioMek ELISA plate reader. Plates were washed between each incubation step with 300 uL per well of PBS containing 0.05% Tween 20 using an automated plate washer. Both detection antibody incubations were performed for 1 h at room temperature. Results were analysed using GraphPad Prism. The dilution factor resulting in an OD value of 0.5 after blank subtraction and normalization was defined as the positive sample dilution.

References

Furevik, A., Tunheim, S. H., Heen, V., Klevan, A., Knutsen, L. E., Tandberg, J. I., & Tingbo, M. G. (2023). New vaccination strategies are required for effective control of winter ulcer disease caused by emerging variant strains of Moritella viscosa in Atlantic salmon. *Fish and Shellfish Immunology*, *137*. https://doi.org/10.1016/j.fsi.2023.108784

Thuvander, A., Fossum, C., & Lorenzen, N. (1990). Monoclonal antibodies to salmonid immunoglobulin: characterization and applicability in immunoassays. *Developmental & Comparative Immunology*, *14*(4), 415-423.
